# Sarcopenia, immune-mediated rheumatic diseases, and nutritional interventions

**DOI:** 10.1007/s40520-021-01800-7

**Published:** 2021-02-10

**Authors:** Alfonso J. Cruz-Jentoft, Susana Romero-Yuste, Eugenio Chamizo Carmona, Joan M. Nolla

**Affiliations:** 1 Servicio de Geriatría, Hospital Universitario Ramón y Cajal (IRYCIS), Madrid, Spain; 2 Department of Rheumatology, Complejo Hospitalario Universitario de Pontevedra, Pontevedra, Spain; 3 Department of Rheumatology, Hospital de Mérida, Mérida, Badajoz, Spain; 4 Department of Rheumatology, Hospital Universitario de Bellvitge, Barcelona, Spain

**Keywords:** Sarcopenia, Immune-mediated rheumatic diseases, IMRDs, Inflammation, Rheumatoid arthritis, Biological disease-modifying antirheumatic drugs, bDMARDs, Nutritional interventions

## Abstract

**Introduction:**

Sarcopenia is defined by a loss of muscle mass and function associated with mortality, decreased physical performance, falls, and disability. Since chronic inflammation and decreased physical activity are risk factors for developing sarcopenia, it is critical to assess the role of sarcopenia in immune-mediated rheumatic diseases (IMRDs). Moreover, nutritional interventions are emerging as key modifiable and affordable options to improve physical performance in sarcopenia.

**Objective:**

The aim of this review is to critically summarize current information on the evidence linking nutritional interventions and sarcopenia in IMRDs.

**Methods:**

The search and selection of articles was performed in Medline, Dimensions.ai, Google Scholar, Cochrane Library, Epistemonikos, and Trip Database. The results were clustered into three areas: sarcopenia and IMRDs, sarcopenia and biological disease-modifying antirheumatic drugs (bDMARDs), and nutritional interventions for sarcopenia.

**Findings:**

Several cross-sectional studies have shown a higher prevalence of sarcopenia in IMRDs, such as rheumatoid arthritis. Although not fully established, evidence linking sarcopenia and other IMRDs (ankylosing spondylitis and systemic sclerosis) has been also described. For secondary sarcopenia prevention and treatment, bDMARDs’ administration proved efficacy in patients with rheumatoid arthritis. Furthermore, there is growing evidence linking nutrition to the prevention and treatment of sarcopenia. Evidence linking unfavourable results in nutritional risk assessment, insufficient intake of protein, vitamin D, antioxidant nutrients, and long-chain polyunsaturated fatty acids and sarcopenia have been reported.

**Conclusion:**

Given that sarcopenia and IMRDs have strong links, further research is needed to improve patient care.

## Introduction

Sarcopenia is a generalized musculoskeletal disorder characterized by loss of muscle mass and function together with decreased physical performance. Loss of muscle mass and function leads to a higher risk of falls and hospitalization rates, decreases the ability to perform activities of daily living, increases functional impairment, worsens quality of life, and increases morbidity [1–3].

Sarcopenia is highly prevalent in older patients and is an important factor contributing to frailty and disability, generating a significant social and economic burden [4]. Sarcopenia prevalence in the community is highly variable (3–24%) as a result of the range of diagnostic criteria and definitions used [5].

Apart from age, sarcopenia-related risk factors include nutritional deficiencies, sedentarism, decreased protein synthesis and regeneration, inflammation, and hormonal and cytokine imbalance, among others [6, 7].

The definition, diagnosis, and assessment of sarcopenia is well described in the literature. The EWGSOP (European Working Group on Sarcopenia Older People) definition of sarcopenia put muscle strength to the forefront, as it is recognised that strength is better than mass in predicting adverse outcomes. In addition, it recommends an algorithm for case finding, diagnosis, and determination of the severity of sarcopenia [2, 3, 8, 9].

## Methods

The literature search for this scoping review was conducted in the following databases since 2014 and in English: Medline, Dimensions.ai, Google Scholar, and Cochrane Library and the evidence-based medicine repositories: Epistemonikos and Trip Database, with the search completion date 11/29/2019. Search strings were performed on the above databases constructed from the term nutritional interventions and/or immune-mediated rheumatic diseases and/or sarcopenia and all their equivalents or synonyms as input terms into the MeSH (Medical Subject Headings) database, the NLM (National Library of Medicine) controlled vocabulary thesaurus. For screening, a restriction was made to those papers included older adults with sarcopenia and diagnosis immune-mediated rheumatic diseases (specifically rheumatoid arthritis, ankylosing spondylitis, and systemic sclerosis). A secondary manual literature search of the selected studies was also conducted to detect possible omissions that could be of interest. Articles were selected in pairs and reviewed in full text to give a detailed view of this complex field. The results were clustered into three areas: sarcopenia and immune-mediated rheumatic diseases (IMRDs); sarcopenia, immune-mediated rheumatic diseases and biological disease-modifying antirheumatic drugs (bDMARDs); and nutritional interventions for sarcopenia.

## Sarcopenia and immuno-mediated rheumatic diseases

IMRDs are accompanied by inflammation, especially joint inflammation, causing pain, joint dysfunction, and destruction, decreased physical activity and quality-of-life impairment. Since decreased physical activity and chronic inflammation are risk factors for sarcopenia, it seems relevant to assess the prevalence of sarcopenia and its association with inflammatory markers, and rheumatic disease course and activity, among others, in patients with IMRDs.

### Inflammatory markers in sarcopenia

Chronic inflammation is a risk factor for developing sarcopenia, because it facilitates muscle catabolism [10]. It is not clear which serum inflammatory molecules are associated with loss of muscle mass and strength and physical performance and which, as such, may be candidates for sarcopenia biomarkers [11].

A cross-sectional study exploring neuromuscular, peripheral pro‐inflammatory, and oxidative stress molecules as potential biomarkers associated with sarcopenia in old-aged people with hip fracture only found differences in peripheral TNF‐α levels and catalase activity. Probably, TNF‐α and catalase are markers of an early inflammatory reaction that is impaired in subjects with sarcopenia [12].

A randomized-controlled trial (RCT) published in 2019 evaluated the association between body composition and inflammatory markers in 1,121 healthy individuals between 65 and 79 years old. It found that muscle mass [assessed by skeletal muscle mass index (SMI): appendicular skeletal muscle mass (ASMM)/height^2^] negatively correlates with proinflammatory serum levels of C-reactive protein (CRP), leptin, and alpha-1-acid glycoprotein (AGP), and positively correlates with Ghrelin, in both men and women [13].

High levels of CRP have also been observed in sarcopenia patients in several cross-sectional studies. A systematic review and meta-analysis conducted in 2016 observed a correlation between significantly higher levels of CRP and sarcopenia (defined as muscle mass loss) [11]. In a cross-sectional study, Can et al. [14] showed that patients with sarcopenia (defined by EWGSOP criteria) had higher levels of CRP and adiponectin and a higher erythrocyte sedimentation rate (ESR). ESR was independently associated with sarcopenia (OR = 1.156; 95% CI 1.057–1.263; *p* = 0.001). In another cross-sectional study, van Atteveld et al. [15] also determined significant inverse correlations between ESR and muscle mass [relative skeletal muscle mass (RMM): skeletal muscle mass (SMM)/weight], muscle strength (grip strength and chair stand test), and physical performance [normal walking speed and timed up and go (TUG)]. Moreover, they observed an association between lower albumin levels and lower muscle strength and physical performance. Otzurk et al. [16] performed a cross-sectional study and observed higher ESR and higher CRP levels in patients with sarcopenia (defined by EWGSOP criteria) than in controls. These authors also analysed the neutrophil/lymphocyte ratio (NLR) and observed an independent association between NLR and sarcopenia (OR = 1.31, 95% CI 1.06–1.62, *p* = 0.013).

A cross-sectional study included a multivariate analysis of 30 serum inflammatory markers in patients with sarcopenia and frailty, and observed a specific pattern of inflammatory markers, with higher levels of CRP, and lower levels of IL-8, myeloperoxidase (MPO), monocyte chemoattractant protein 1 (MCP1), and platelet-derived growth factor BB (PDGF-BB). This study also showed gender-specific pattern differences: for example, women had higher levels of selectin P and eotaxin, while men had lower levels of IFN-γ, IL-17, and TNF-α, among others [17].

Finally, CRP and IL-8 levels and adiponectin/leptin ratio may have prognostic value for the development of sarcopenia. A longitudinal study that included 336 adults aged 59–70 years of age, with an average follow-up period of 10.8 years, observed significant associations between higher levels of CRP, higher levels of IL-8, and a lower adiponectin/leptin ratio at baseline and the development of sarcopenia (defined by EGWSOP) at the follow-up visit [18].

### Sarcopenia and rheumatoid arthritis

Loss of muscle strength in patients with RA may be seen frequently. Longer disease duration and higher disease activity should lead to development of sarcopenia due to chronic inflammation [19].

Several cross-sectional studies have shown a higher prevalence of sarcopenia in patients with rheumatoid arthritis (RA) compared to healthy controls [20–25]. The prevalence of sarcopenia described in studies in patients with RA ranges from 7.8% [26] to 87.5% [24]. One of the reasons for these differences is the different criteria and assessment methods for sarcopenia used in each study. Even so, it should be noted that in a cross-sectional study by Tournadre et al. [26] found the same sarcopenia prevalence in RA patients (low, 7.8%) using both the EWGSOP definition (muscle mass and function) and muscle mass alone (SMI: ASMM/height^2^).

Several studies have analysed factors associated with the development of sarcopenia in patients with RA. Although not fully established, these include, but are not limited to, age, sex, nutritional status, disease activity, and degree of disability.

#### Gender

Several studies have shown gender differences in the prevalence of sarcopenia in RA patients [23, 27]. In a cross-sectional study, Baker et al. [27] found that muscle mass [measured with appendicular lean mass index (ALMI): ALM/height^2^] was lower in men than in women (*p* < 0.0001) and they observed a 3–8 times higher probability of sarcopenia in men in one of the two study cohorts. Male sex was also associated with the development of sarcopenia in the correlation research of sarcopenia, skeletal muscle, and disease activity in RA [28].

#### Nutritional status

Although body mass index (BMI) has been associated with sarcopenia in previous publications [28–32], many studies show that muscle mass loss is not always associated with lower BMI. The low or no weight loss in these patients is explained by a stable or slightly increased fat mass, i.e., sarcopenic obesity. The concept of sarcopenic obesity is still an evolving one and has not been well studied in rheumatic diseases [33]. One observational study evaluated sarcopenia (defined by SMI) in women with RA vs. healthy controls and concluded that despite having a similar BMI, the prevalence of sarcopenia was significantly higher in patients with RA [20]. Accordingly, one transversal study in men and women with RA correlated sarcopenia (defined by SMI) to normal BMI (OR = 82.1, 95% CI 3.8–1733.3; *p* = 0.005) and over fat BMI (OR = 12.3; 95% CI 2.27–67.6; *p* = 0.004) [21]. A cross-sectional study by Vlietstra et al. [34] observed an association between sarcopenia (defined according to muscle mass) and higher fat content (OR = 1.1, 95% CI 1.0–1.2; *p* < 0.02), and use of glucocorticoids (OR = 1.08; 95% CI 1.0–1.2; *p* = 0.017) in patients with RA.

#### Disease activity

The relationship between the presence of sarcopenia and RA activity or severity is not clearly established. Some studies have found no association between sarcopenia and markers of disease activity such as Disease Activity Score 28 (DAS28) [20, 21, 32, 35]. In a cross-sectional study, Barone et al. [35] observed a prevalence of sarcopenia (muscle mass and strength) of about 20% in patients with RA, but no relationship with RA activity. In contrast, disability and age were positively associated in this study with sarcopenia.

Other studies in RA have positively correlated disease duration and activity with sarcopenia. In a longitudinal study of 294 RA patients, Park et al. [36] showed that sarcopenia (defined by SMI) has a prognostic value for increased disease activity (DAS28-ESR) at 3 years (OR = 4.477; 95% CI 1.661–12.067; *p* = 0.003). Another cross-sectional study in women with RA observed an association between inflammation levels and long-standing RA (CRP levels) and muscle mass (ALMI) [37]. In a case–control study, Reina et al. [30] found that BMI, SMI, and lean appendicular mass are inversely correlated, and that fat mass is directly correlated with disease duration in patients with RA, in both men and women. A cross-sectional study of 388 women with RA observed that disease duration (OR = 1.06; 95% CI 1.04–1.09), joint damage (Steinbrocker’s class, OR = 3.19, 95% CI 1.60–6.53), and age (OR = 1.64; 95% CI 1.26–2.17) were independent factors positively associated with sarcopenia [defined by muscle mass, strength, and function, according to the Asian Working Group on Sarcopenia (AWGS) definition]. However, bDMARDs use (OR = 0.51; 95% CI 0.28–0.93) and a good nutritional status (OR = 0.61; 95% CI 0.51–0.71) were negatively associated with sarcopenia [31].

Other studies have also observed an association between sarcopenia (defined by muscle mass, SMI) and radiographic joint damage (OR = 2.154; 95% CI 1.032–4.497; *p* = 0.041) [38] and bone erosion (OR = 0.057; 95% CI 0.006–0.532; *p* = 0.012) in patients with RA [21].

#### Disability and falls

A cross-sectional study of 240 patients with RA showed no significant relationship between a disability index or RA activity (determined by DAS28-ESR) and sarcopenia (defined by AWGS). Factors associated with sarcopenia in this study were age (OR = 1.08, *p* = 0.008), BMI (OR = 0.73, *p* < 0.001), CRP (OR = 1.76, *p* = 0.017), and hip bone mineral density (BMD) (OR = 0.61, *p* = 0.037) [32].

CHIKARA is a prospective, observational study that included 100 patients of both sexes with RA. Early analysis showed an independent correlation between sarcopenia (defined by the AWGS definition) and high body fat mass, low BMI, and high matrix metalloproteinase-3 (MMP3) [29]. In a subsequent study, univariate analysis observed that male sex, old age, glucocorticoid use > 5 mg/day, high levels of MMP3, and higher disability evaluated by the Health Assessment Questionnaire (HAQ) were associated with sarcopenia (defined by AWGS) development at 2 years in RA patients [28].

Sarcopenia is a risk factor for fall events. In a cross-sectional study, Vlietstra et al. [34] showed a weak association between sarcopenia (defined by muscle mass) and fatigue in patients with RA. Nevertheless, the analysis of CHIKARA data at 1 year did not show a significant relationship between the risk of fall events and sarcopenia (defined by AWGS). No relationship was observed between the risk of fall events and disease activity and CRP; but height and obesity levels showed a negative correlation with fall events [39].

#### Bone mineral density

Decreased muscle mass may appear together with decreased bone mass, in what has been named osteosarcopenia [40, 41], but the relationship of sarcopenia, low BMD, and osteoporosis in RA patients is not fully established understood [42].

Confavreux et al. [43] revised the systemic bone effects of biologic therapies in IMRDs (such as RA and ankylosing spondylitis). Feklistov et al. [22] observed osteoporosis (low BMD) in a cross-sectional study among 48% of women with RA versus 45% in healthy controls. Sarcopenia (muscle mass, strength, and physical performance) was observed in 25% of RA patients, and osteosarcopenia (sarcopenia + low BMD) was found in 15%, versus 12.5% and 5% in healthy controls, respectively. TOMORROW study was a prospective cohort that shows data from 208 patients with RA and 205 age- and sex-matched healthy controls [44, 45]. Okano et al. [44] showed lower muscle mass and lower BMD in patients with RA, and a positive correlation between both parameters. In a subsequent subanalysis of this cohort, Inui et al. analysed muscle mass and BMD in individuals > 65 years for a period of 7 years, and observed that muscle mass [determined by appendicular skeletal mass index (ASMI)] is an independent factor associated with BMD change (*p* = 0.0020) in patients with RA but not in healthy controls [45].

#### Frailty

Sarcopenia and frailty, defined as physical and cognitive function deterioration in older adults, often overlap [46]. Early detection of frailty and its risk factors, such as sarcopenia, is important for prevention and management. In a cross-sectional study of 282 patients with AR, prevalence of frailty was as high as 21.5%, and 42% of them were older than 65 years. Overall, 31% of patients had sarcopenia (defined by SMI) [47]. In a cross-sectional analysis of CHIKARA study data at 1 year, Tada et al. [48] observed a positive association between sarcopenia (defined by AWGS) and frailty (OR = 3.1; 95% CI 1.2–1.8; *p* < 0.024). Observed frailty and pre-frailty prevalences were 18.9% and 38.9%, respectively. In these groups, sarcopenia prevalence was 39% and 41%, respectively, and among patients without frailty, it was 18%. Other risk factors were MMP3 levels, age, disease severity, and joint dysfunction, whilst RA treatment was associated negatively with the onset of sarcopenia. Finally, a cross-sectional study of 210 patients with RA and 100 healthy controls found a higher prevalence of frailty among RA patients, showing 16.6% with frailty and 32.4% with pre-frailty. Risk factors associated with frailty were age (OR = 1.12; 95% CI 1.07–1.17; *p* < 0.0001), comorbidities (OR = 1.51; 95% CI 1.01–2.27; *p* = 0.0446), and disease activity (OR = 1.10; 95% CI 1.04–1.16; *p* = 0.0006) [49].

### Sarcopenia and ankylosing spondylitis

Ankylosing spondylitis (AS) causes decreased bone mass, stiffness, and movement loss, which may be related to loss of muscle mass and the development of sarcopenia. However, the prevalence and impact of sarcopenia in AS patients has not yet been clearly established [35, 50].

A cross-sectional by Ibáñez et al. [51] observed a decrease in muscle mass (and fat mass) associated with disease activity in male patients with AS, but not in women with AS. In both men and women, disease activity [Ankylosing Spondylitis Disease Activity Score (ASDAS) CRP] correlated negatively with fat mass. In a cross-sectional study of 10 patients with AS and 10 healthy controls, Røren et al. [52] observed significantly lower appendicular lean body mass (but no total mass), lower muscle strength, and a reduced number of type II muscle fibres in patients with AS.

A cross-sectional carried out by El Maghraoui et al. [53], which included 67 males with AS and 67 healthy controls, observed lower muscle mass in patients with AS. Prevalences of pre-sarcopenia, sarcopenia (defined by EWGSOP), and cachexia in patients with AS were 50.4%, 34.3%, and 11.9%, respectively. Sarcopenia and cachexia were significant associated with higher disease activity (Bath Ankylosing Spondylitis Disease Activity Index [BASDAI]) and lower BMD. Finally, in a cross-sectional study by Barone et al. [35] also observed a high prevalence of sarcopenia among patients with AS, around 20%.

### Sarcopenia and systemic sclerosis

Several studies have observed a high prevalence of sarcopenia among patients with systemic sclerosis (SS), higher than that of healthy individuals [35, 54–57].

In a large cohort of 141 patients with SS, the prevalence of sarcopenia (defined by SMI) was around 20.7%. Importantly, sarcopenia prevalence in malnourished patients was significantly higher [54]. In a pilot intervention study of 18 patients with SS and gastrointestinal involvement, the percentage of patients with sarcopenia (defined by muscle mass) was lower after 6-week nutritional intervention (54–39%, *p* < 0.02) [55].

However, the association between sarcopenia and disease duration in patients with SS has not been established [54, 56, 57].

These studies show an interesting relationship between IMRDs and sarcopenia, but are burdened by differences in sarcopenia-related terms definitions, especially those that only evaluate muscle mass, and those that use a modern definition of sarcopenia.

## Sarcopenia, immune-mediated rheumatic diseases, and biological disease-modifying antirheumatic drugs

Treatment of IMRDs may have an impact on sarcopenia. However, only a few studies have reported on the links between drugs and sarcopenia in this setting.

The deleterious effects of glucocorticoid (GC) on muscles have been well described elsewhere [58]. Patients with IMRDs using GC are at high risk for fractures, due to both the direct and indirect negative effects of GC on bone mass, and bone and muscle strength, and due to activity of the underlying inflammatory disease [59]. In a cross-sectional analysis of CHIKARA in patients with RA, GC use was more frequent among patients with sarcopenia than in patients without sarcopenia. Univariate analysis showed that GC dose was significantly associated with sarcopenia onset [60]. However, few data are available on other drugs used to treat IMRDs.

A prospective cohort study by Hasegawa et al. [61] evaluated bDMARDs’ activity on sarcopenia in men and women with RA starting bDMARDs treatment for the first time. bDMARDs used in the study included certolizumab pegol (24.4%), adalimumab (17.1%), abatacept (17.1%), golimumab (14.6%), tocilizumab (12.2%), infliximab (7.3%), and etanercept (7.3%). After 6 months of treatment with bDMARDs, physical activity, nutritional status, and quality of life significantly improved, and disease activity also was significantly reduced. Although muscle mass did not increase notably, the proportion of patients with sarcopenia tended to decrease. In view of these data, the authors suggested that bDMARD administration may be useful for secondary sarcopenia prevention in patients with RA. Similarly, in a cross-sectional study by Torii et al. [31] observed a negative association between bDMARDs use and sarcopenia in women with RA.

Briot et al. [62] in a prospective open study analysed the effect of anti-TNF-α treatment in male and female patients with spondyloarthropathy. Drugs used in this study included infliximab (89.5%) and etanercept (10.5%). After 1 year of treatment, significant increases in body weight, BMD, bone markers, and IGF-1 were observed. Therefore, treatment with anti-TNF-α in AS improves bone resorption together with an increase in body weight, lean body mass, and IGF-1.

These limited data regarding bDMARDs therapy suggest that this treatment has the potential to improve sarcopenia. Hence, future studies exploring this outcome are of the outmost interest.

## Nutritional interventions for sarcopenia

There is growing evidence linking nutrition to muscle mass, strength, and function, suggesting its important role in both the prevention and treatment of sarcopenia [63]. Appropriate quality dietary patterns that ensure sufficient intake of protein, vitamin D, antioxidant nutrients, and long-chain polyunsaturated fatty acids are key modifiable and affordable interventions to improve physical performance in older people and/or in patients with particular diseases, such as frailty or sarcopenia [64–66].

A systematic literature review evaluated the association between sarcopenia and nutritional status, and observed a relationship between sarcopenia and a poor nutritional status [67].

In another systematic review evaluated the quality of the diet and sarcopenia, studying muscle mass, strength and physical performance, and sarcopenia risk. Evidence showing a relationship between “healthier” diets and better results in muscle mass and strength was low. In contrast, a relationship between “healthier” diets and a lower risk of decreased physical performance and a reduced risk of sarcopenia were observed. This study, then, shows benefits associated with “high quality” diets for physical performance improvement in older patients [68].

An observational study called SarcoPhAge study assessed micronutrient and macronutrient intake in patients of both sexes with sarcopenia. Study results suggest an association between an unbalanced diet and sarcopenia and poor musculoskeletal health, although further prospective studies are needed to confirm these findings [64]. Current guidelines on sarcopenia management consider the role of nutritional intervention on sarcopenia, but overall evidence is weak [69].

### Proteins

Dietary proteins provide essential amino acids for muscle protein synthesis, and of these, leucine is especially important. Moreover, dietary proteins may act as an anabolic trigger, playing a key role in muscle protein synthesis [66, 70]. Protein turnover is key to balancing catabolism and anabolism and to maintaining muscle mass balance. Several studies show that an adequate high-quality protein intake is essential to maintain muscle mass. However, studies linking protein/amino acid intake to muscle strength and mass and function show inconsistent results [65].

A critical review by Hickson [70] found that trials performed with complete protein supplementation did not show a consistent effect on muscle mass, strength, or function. This could be explained by differences in study design, protein supplement composition and failure to monitor voluntary food intake, compliance, and baseline nutritional status. However, a systematic review by Shad et al. [71] showed that administrating aminoacid/protein to young and old individuals induces muscle protein synthesis (MPS). Moreover, the amino acid/protein dose and leucine content should exceed a certain threshold to stimulate equivalent MPS rates in young and older adults. Below this threshold, age-related muscle anabolic resistance is observed.

A systematic review and meta-analysis evaluated the efficacy of dairy proteins in sarcopenia-related functions in middle-aged and older adults. Dairy protein supplementation significantly increased appendicular muscle mass in middle-aged and older adults, although it showed no effect on improving muscle strength [72]. Finally, a systematic review analysed the effects of leucine or leucine-enriched protein (range 1.2–6.0 g leucine/day) supplementation in sarcopenic patients. Results showed that leucine administration improved sarcopenia by improving lean muscle mass. The effect on muscle strength showed mixed results, and the effect on physical performance has been little studied [73].

In brief, there is significant evidence of the importance of protein intake as the main stimulus for muscle protein synthesis and for maintaining muscle mass and strength in old age. Several expert groups have proposed an increase in dietary protein recommendations for older age groups to 1.0–1.2 g/kg body weight per day. However, more studies are needed to understand the specific benefit of a high-protein diet on physical function [66].

### Vitamin D

Muscle mass loss and vitamin D deficiency often occur concomitantly, and are linked to weakness, fall events, and frailty in older patients [66]. Vitamin D receptors are widely expressed in muscle cells; however, their expression decreases with age, contributing to sarcopenia. Indirectly, vitamin D regulates calcium levels in the muscle and muscle fibre atrophy. Vitamin D deficiency causes muscle weakness that can be reversed with external vitamin D administration. Several studies associate muscle mass with vitamin D levels, although this relationship has not been fully clarified [65, 74].

It is difficult to assess the role of vitamin D, because there are no studies of vitamin D alone without protein supplementation. An RCT named The PROVIDE study evaluated vitamin D and leucine-enriched diets in older adults with sarcopenia. After 13 weeks, an improvement in muscle mass and lower limb function was observed in sarcopenic patients, highlighting the value of nutritional supplementation among these patients [75]. In an RCT carried out by Bo et al. [76] also showed that combined supplementation with protein, vitamin D, and vitamin E can significantly improve muscle mass [relative skeletal mass index (RSMI)], muscle strength and anabolic markers such as IGF-I and IL-2 in older adults with sarcopenia.

In short, we found significant evidence of the benefits of vitamin D supplementation, when included in multicomponent oral nutritional supplements, in maintaining muscle mass, strength, and physical function in old age, and in preventing and treating sarcopenia [66].

### Omega-3 fatty acids

It has been suggested that the anti-inflammatory properties of ω-3 fatty acids (ω-3) are beneficial for muscle mass, strength, and function, and that they can prevent the low-grade, age-related chronic inflammation that contributes to the development of sarcopenia. Although the mechanisms by which ω-3 exert their effect on muscle mass and function are still unclear, a growing number of studies demonstrate the potential beneficial effect of dietary supplementation with ω-3 in older sarcopenic individuals [77]. A review found that ω-3 supplementation seems to increase muscle mass and prevent muscle catabolism independent of anabolic stimuli or anti-inflammatory effects in patients with primary and secondary sarcopenia. However, one of the included studies failed to show any effect of supplementation on muscle mass [78].

### β-Hydroxy-β-methylbutyrate

β-Hydroxy-β-methylbutyrate (HMB) has been shown to reduce protein degradation, increase protein synthesis, and increase cholesterol production in muscle cells, conferring more stability to cell membranes. Moreover, HMB is a leucine metabolite, and 5–10% of ingested leucine is converted into HMB. HMB administration has shown benefits in muscle mass loss, strength, and function in several studies [70].

In an RCT conducted by Cramer et al. [79] evaluated high-protein oral + HMB nutritional supplementation in malnourished adult patients. Nutritional supplementation improved strength outcomes in malnourished older patients with sarcopenia. In patients with mild–moderate sarcopenia, nutritional supplementation + HMB improved strength and muscle quality in lower limbs compared to controls.

Oktaviana et al. [80] performed a systematic review to determine HMB effects on sarcopenic or fragile individuals. The results showed increased lean body mass and preserved muscle strength and function after HMB supplementation. The main limitation was the reduced number of currently available studies with HMB. In this respect, the effect of HMB supplementation on the mass, strength, and muscle function of older people with sarcopenia or frailty may be underestimated. A review evaluated the effect of oral HMB-enriched protein-rich nutritional supplements and found that they mitigated the decline of muscle mass and preserved muscle function, especially during hospital rehabilitation and recovery [81].

Therefore, more randomized clinical studies evaluating HMB administration in different clinical settings are needed to determine the benefits of supplementation.

### Other micronutrients

It has been suggested that the antioxidant elements (vitamins C, E, and carotenoids and trace elements: Cu, Mn, Se, and Zn) intervene in muscle mass and strength, while minerals (Mg) intervene in muscle function and performance, and particular biocomponents, such as phenols, in muscle strength and mass [65].

The SarcoPhAge study assessed the micronutrient and macronutrient intake of patients of both sexes with sarcopenia. The adjusted analysis showed that sarcopenic patients consumed significantly lower amounts of two macronutrients (protein, lipids) and five micronutrients (potassium, magnesium, phosphorus, iron, and vitamin K) compared to non-sarcopenic participants (*p* < 0.005) [64].

### Combined nutritional interventions

A systematic review evaluated the effect of nutritional intervention combined with physical activity on muscle mass and function in individuals over 60 years of age. Physical activity affected muscle mass and function positively; however, the results of interactive effects with the nutritional intervention were limited [82]. A systematic review evaluated the effect of nutritional intervention, physical activity, and the combined effect. The results highlight the importance of physical exercise (with and without concomitant nutritional interventions) for improving physical performance in patients with frailty and sarcopenia. In these patients, muscle strength improved with multidisciplinary treatment and physical exercise [83].

### Immune-mediated rheumatic diseases and nutritional interventions

Nutritional abnormalities are prevalent in patients with IMRDs, and affect prognosis, quality of life, autonomy, independence, and even mortality. The aetiology of nutritional alterations is multifactorial, and malnutrition can be associated with chronic inflammatory processes (cachexia), acute inflammatory processes (protein-calorie malnutrition), and low food intake [84].

Nutrition plays an important role in both the progression and clinical outcomes of inflammatory diseases such as RA. Although the effect of nutrition on musculoskeletal diseases is not well studied, several clinical studies have linked supplementation with fatty acids and probiotics, and anti-inflammatory diets with improved symptoms and activities of daily living in patients with RA [85]. One case–control study in patients with RA showed that oral administration of creatine improves muscle mass, but no effect on muscle strength or function was observed [86]. The integrated management of IMRDs should include prevention, identification, and management of nutritional disorders [84].

Hugo et al. [87] performed an observational study to evaluate energy expenditure and nutritional complications in RA patients with metabolic syndrome and rheumatoid cachexia. They found that low levels of physical activity and GC use are associated with nutritional complications in patients with RA, suggesting a potential strategy for therapeutic intervention.

## Conclusions and future research directions

The link between sarcopenia and rheumatoid diseases is an interesting growing area of study; however, it requires more in-deep studies (Table 1). To better understand the interaction between these two groups of diseases and its potential treatment, geriatricians and rheumatologists need to work closely. Finally, it would be helpful to use modern definitions of sarcopenia, and not just muscle mass definition, to get comparable and universal results.

**Table 1:** Some gaps and research priorities

1	Most available data are based on rheumatoid arthritis, and more studies on other IMRDs are needed
2	IMRDs may help basic science to better understand the role of inflammation in the pathophysiology of sarcopenia
3	There are hints that treatment of IMRDs with bDMARDs may have an impact on sarcopenia, and this should be explored in trials of available and new drugs for IMRDs
4	bDMARDs may have some potential to be explored in the treatment of sarcopenia
5	More studies are needed on nutritional interventions for IMRDs
6	Comparison studies on handgrip strength and other muscle strength measures may help understanding the differential role of hand inflammation and muscle function in this diagnostic test
